# Associations between Low-Carbohydrate Diets and Low-Fat Diets with Frailty in Community-Dwelling Aging Chinese Adults

**DOI:** 10.3390/nu15143084

**Published:** 2023-07-09

**Authors:** Xiaoxia Li, Qingan Wang, Linfeng Guo, Yixuan Xue, Yuanyuan Dang, Wanlu Liu, Ting Yin, Yuhong Zhang, Yi Zhao

**Affiliations:** 1 School of Public Health, Ningxia Medical University, Yinchuan 750004, China; lixiaoxiajay2023@outlook.com (X.L.);; 2 NHC Key Laboratory of Metabolic Cardiovascular Diseases Research, Ningxia Medical University, Yinchuan 750004, China; 3 Key Laboratory of Environmental Factors and Chronic Disease Control, Ningxia Medical University, Yinchuan 750004, China

**Keywords:** frailty index, elderly, macronutrients, protein, carbohydrate, fat

## Abstract

Frailty is a major health issue associated with aging. Diet affects frailty status; however, studies on the associations between the low-carbohydrate diet (LCD) score, low-fat diet (LFD) score and frailty in older Chinese adults are scarce. This study aimed to examine the associations between the LCD score, LFD score and risk of frailty in older Chinese adults. We analyzed data from 6414 participants aged ≥ 60 years from the China Northwest Natural Population Cohort: Ningxia Project. Frailty was measured using the frailty index (FI), calculated from 28 items comprising diseases, behavioral disorders and blood biochemistry and classified as robust, pre-frail and frail. LCD and LFD scores were calculated using a validated food frequency questionnaire (FFQ). Multiple logistic regression models were used to evaluate associations between LCD, LFD scores and frail or pre-frail status after adjusting for confounders. Participants’ mean age was 66.60 ± 4.15 years, and 47.8% were male. After adjusting for age, sex, educational level, drinking, smoking, BMI, physical activity and total energy, compared to the lowest quartile (Q1: reference), the odds ratios (ORs) for pre-frail and frail status in the highest quartile (Q4) of LCD score were 0.73 (95% confidence intervals: 0.61–0.88; *p* for trend = 0.017) and 0.73 (95%CI: 0.55–0.95; *p* for trend = 0.035), respectively. No significant associations were observed between LFD score and either pre-frail or frail status. Our data support that lower-carbohydrate diets were associated with lower pre-frail or frail status, particularly in females, while diets lower in fat were not significantly associated with the risk of either pre-frail or frail status in older Chinese adults. Further intervention studies are needed to confirm these results.

## 1. Introduction

As lifespan extends globally, frailty has become a major health concern associated with population aging [1]. Frailty is characterized by declining functioning across multiple physiological systems, accompanied by an increased risk of health problems [1,2]. Currently, the frailty index (FI) is a cumulative score based on identified health status deficits that is frequently used to describe frailty status in different older populations [3]. Several epidemiological studies have demonstrated that FI is related to mortality [4,5], cardiovascular disease [6], diabetes [7] and obesity [8].

Diet intake is a key modifiable factor potentially related to frailty status [9]. The results from a longitudinal study of 13,721 older Chinese adults found that the dietary diversity score was associated with a lower risk of FI [10]. Diet quality scores were significantly associated with FI among 15,249 US participants [11]. Different dietary patterns also influence the frailty state. For example, the Mediterranean diet was protective in maintaining a lower FI [12]; three dietary patterns summarized based on Japanese older adults, including salt and pickles, sugar and a fat- and protein-rich dietary pattern, were associated with FI [13]; the results from Chinese older adults also found that the egg–bean–pickle–sugar pattern and fruit–vegetable–meat–fish pattern reduced frailty incidence [14]. A recent study demonstrated that dietary carbohydrates were positively associated with increased frailty risk in Baltimore participants [15]; thus, lower carbohydrate consumption may prevent frailty. The low-carbohydrate diet (LCD) score, with lower daily carbohydrate and higher protein and fat intake, has been associated with mortality [16], diabetes [17], cognition in the elderly [18] and weight loss [19]. A 15-year follow-up study of 1210 French individuals suggested that higher simple carbohydrate intake was significantly associated with incident frailty risk, but the LCD score was not. Notably, frailty was described using the frailty phenotype, which focuses on five physical factors, including unintentional weight loss, self-reported exhaustion, weakness (grip strength), slow walking speed and low physical activity, and not FI, which emphasizes accumulated health deficits [20]. Although low carbohydrate intake has been suggested as a frailty prevention strategy, currently relevant studies are limited to certain Western countries, such as the US [15] and France [20]. The results regarding the association of the LCD score with frailty risk are still considerably lacking in the Chinese context. It is well known that dietary intake and habits in Asian countries differ substantially from Western countries. In China, although the percentage of energy from carbohydrates has decreased over the past 20 years (from 62.6% to 50.6%), energy from the intake of low-quality carbohydrates derived from refined grains remains challenging to reduce (about 36.2%); furthermore, the estimated energy intake from total fat has significantly increased to 35.8%, above the recommended value of Chinese dietary guidelines [21]. However, few related studies using the LCD score, which combines low-carbohydrate and high-fat diets, have examined the long-term associations of carbohydrates with frailty among older Chinese adults. In addition, it is unclear whether adherence to low-fat diets (LFDs), which are low in total fat, especially unhealthy fat, is associated with lower frailty. Furthermore, evidence regarding the potential associations between LFD and LCD scores with pre-frailty is limited.

Consequently, in this study, we aimed to explore the associations of LCD and LFD scores with frailty and pre-frailty in older participants from rural areas of northwest China. We hypothesized that higher LCD and LFD scores would be associated with a lower risk of frailty and pre-frailty.

## 2. Materials and Methods

### 2.1. Study Population

This study was derived from the baseline of the China Northwest Natural Population Cohort, Ningxia Project (CNC-NX), an ongoing population-based prospective cohort study. Details on the study design have been published elsewhere [22]. Briefly, 15,802 participants (age range of 35–74 years) from Wuzhong and Shizuishan cities in the Ningxia Hui Autonomous Region of China were enrolled at baseline between March 2018 and August 2019. Demographic characteristics, food frequency questionnaires (FFQs) and biochemical measurements were obtained from all participants. Of these 15,802 participants, we first included 6863 participants aged ≥ 60 years. We further excluded those with incomplete data from BMI or WHR (*n* = 240), missing blood pressure data (*n* = 109) or extreme dietary energy intake, such as >4200 or <600 kcal/day for males (*n* = 43) and >3600 or <500 kcal/day for females (*n* = 57) [23]. Finally, 6414 participants were included as the final analytic sample in our study, including 2205 participants aged 60–64 years (34.4%), 2602 participants aged 65–69 years (40.5%), 1403 participants aged 70–74 years (21.9%) and 204 participants aged 75 years and older (3.2%). This study was approved by the ethics committee of Ningxia Medical University (No.2018-012), and all participants provided written informed consent.

### 2.2. General Information and Anthropometric Measurement

General characteristics (e.g., sex, age, educational level, cigarette smoking, alcohol intake and disease history) were collected via face-to-face interviews using questionnaires by trained research assistants. Body mass index (BMI), waist circumference (WC) and waist-to-hip ratio (WHR) were estimated using bioelectrical impedance analysis (BIA) devices (InBody 370 system, Biospace, Seoul, Republic of Korea). Systolic blood pressure (SBP) and diastolic blood pressure (DBP) were measured using an OMRON automatic monitor after the participants had rested for at least 5 min.

### 2.3. Biochemical Measurements

Blood samples were collected from the participants between 6:00 and 8:00 a.m., after an overnight fasting, by nurses at the local medical center. Serum samples were separated from whole blood within 2 h in the field. Biochemical indexes, including fasting blood glucose (FBG), serum high-density lipoprotein cholesterol (HDL-C), low-density lipoprotein cholesterol (LDL-C), total cholesterol (TC) and triglycerides (TG), were determined using an automatic biochemical analyzer following standard protocols (Mindray BS-430, Shenzhen, China).

### 2.4. Dietary Surveys and Assessment of LCD and LFD Scores

Diet was assessed using a validated semi-quantitative FFQ, which included 69 food items commonly consumed by Chinese adults and some specialty local foods like eight treasures tea, Naan, etc. Study participants were asked to report their average frequency of consumption of foods throughout the previous year by selecting one of five frequency categories (never or seldom, 1–3 times per month, 1–3 times per week, 4–6 times per week, or per day). The selected frequency category for each food item was then converted to a daily frequency of consumption, and daily intakes of energy, nutrients and foods were calculated by multiplying the frequency of consumption by the nutrient content of the selected portion size. The LCD score was calculated based on the percentages of energy from carbohydrate, fat and protein for each participant. Participants were ranked into 11 strata. For fat and protein, individuals in the highest stratum received 10 points and those in the lowest stratum received 0 points; for carbohydrate, the order of the strata was reversed. Then, the points for the three macronutrients were summed to create the LCD score, ranging from 0 to 30 (Supplement Table S2). We used similar methods to calculate the LFD score (Supplement Table S3) [24].

### 2.5. Definition of FI

The FI was created using 28 variables according to a standard procedure [5,25], including 15 items related to diseases, 10 items related to symptoms/signs and 3 items related to physical measurements (Table S1 in the Supplement). Each variable was categorized as 0–1, where 0 indicated the absence of a deficit and 1 indicated the maximal expression of the deficit. Thus, FI = (No. of health deficits present)/(No. of health deficits measured, *n* = 28), and FI was then divided into three categories: robust (FI ≤ 0.10), pre-frail (FI > 0.10 to < 0.25) and frail (FI ≥ 0.25).

### 2.6. Statistical Analysis

Continuous variables including age, BMI, WC, SBP, DBP, HDL-C, LDL-C, TG, TC and dietary intake were reported as mean ± standard deviation (SD), and analysis of variance was used to compare differences among robust, pre-frail and frail groups. Categorical variables including sex, education level, cigarette smoking and alcohol intake were presented as numbers (%) in the sample. Chi-square tests were used to compare differences among the three FI categories. LCD and CFD scores were divided into quartiles (Q1–Q4) based on the 25th, 50th and 75th percentiles of the score distribution. The median values in each quartile were used to calculate *p* values for trends. Logistic regression was used to estimate odds ratios (ORs) and 95% confidence intervals (CIs) of pre-frail or frail status associated with different levels of LCD and LFD scores, after adjusting for multiple putative confounding factors. We further conducted stratification analysis by age (<65 or ≥65 years), sex (male or female), smoking status (yes or no), drinking (yes or no), BMI (kg/m^2^) (≤23.9, 24.0–27.9, or ≥28.0) and FBG levels (mmol/L) (<5.6, 5.6–6.9, or ≥7.0) for associations between LCD score, LFD score and frailty or pre-frail status. Meanwhile, *p* values for the product terms between each diet score and stratification variables were used to estimate the significance of interactions. A forest plot for subgroup analysis was conducted using R statistical software (version 3.4.2; R Core Team). Other data were analyzed using SPSS software (version 23.0; IBM Corp, Armonk, NY, USA) and *p* < 0.05 was considered statistically significant.

## 3. Results

### 3.1. Characteristics of the Study Participants

Of all 6414 subjects, 3152 (49.1%) were robust, 2475 (38.6%) were pre-frail and 787 (12.3%) were frail. The characteristics of the study subjects were compared among the three groups in Table 1. A gradient increase was observed in BMI, WC, WHR, TG and TC among the three groups (all *p* < 0.01), whereas an opposite trend was observed for HDL-C (*p* < 0.001). Compared to non-frail subjects, those who were pre-frail or frail had higher SBP and DBP (all *p* < 0.05). Additionally, robust individuals had a higher LCD score than both pre-frail and frail individuals; frail individuals had a higher LFD score than pre-frail individuals; and robust participants also had greater energy and protein intake than both the pre-frail and frail groups.

The frailty status and characteristics of study participants across quartiles of the LCD and LFD scores are compared in Table 2. We detected the differences in WC, BMI, SBP, DBP and LDL-C across Q1 to Q4 of the LCD score (all *p* trends < 0.05). A similar pattern of differences was found for WC, WHR, BMI, SBP, TG and LDL-C across the quartiles of the LFD score (all *p* trends < 0.05). For other dietary intake measures, statistically significant differences were observed across quartiles of both the LCD and LFD scores (all *p* trends < 0.001). However, no significant differences in frailty status existed across the Q1 to Q4 groups of the LFD score (*p* trend = 0.607), whereas the differences across the quartiles of the LCD score were statistically significant (*p* trend = 0.003); specifically, the prevalence of pre-frailty and frailty was significantly higher in Q1 compared to Q4 of the LCD score.

### 3.2. Association between LCD and LFD Scores and Pre-Frail or Frail

Table 3 showed that after adjusting for multivariable, the ORs of being pre-frail from the lowest quartiles (Q1) to the highest Q4 of LCD score were 1.00 (ref), 0.88 (95% CI: 0.76–1.03), 0.96 (95% CI: 0.82–1.11) and 0.73 (95% CI: 0.61–0.88) (*p* for trend = 0.017). A similar association was found between LCD score and frailty: the corresponding ORs for frail were 1.00 (ref), 0.65 (95% CI: 0.52–0.82), 0.85 (95% CI: 0.68–1.06) and 0.73 (95% CI: 0.55–0.95) (*p* for trend = 0.035). However, non-significant associations were found between LFD score and risk of pre-frailty (*p* for trend = 0.203) and frailty (*p* for trend = 0.275) after adjusting for covariates.

To further examine the effects of dietary fat quality on frail and pre-frail status, we analyzed the associations of saturated fat (SFA) intake and unsaturated fat intake (MUFA and PUFA) with pre-frailty and frailty. After adjusting for multiple confounding factors, the study found that SFA intake was positively correlated and PUFA intake was negatively correlated with pre-frailty and frailty; however, the results were not statistically significant (Table S4 in the Supplement).

In the stratified analysis, the associations remained persistent in most subgroups. After adjusting for confounding factors, statistically significant interactions were also detected between LCD score and drinking for frailty (*p*  <  0.001 for interaction) and between LFD score and drinking interaction for pre-frailty (*p*  <  0.05 for interaction). Interactions were also found between FBG levels and LFD score for frailty (*p*  =  0.030 for interaction) (Figure S1 in the Supplement). Considering the difference in FI between males and females, we analyzed the association between LCD, LFD scores and frailty according to sex. The results showed that only in females did the highest LCD score have a significantly lower risk of frailty and pre-frailty, the adjusted ORs for pre-frailty and frailty in Q4 of the LCD score were 0.62 (95% confidence intervals: 0.47–0.80; *p* for trend = 0.002) and 0.65 (95%CI: 0.46–0.92; *p* for trend = 0.044), respectively (Table S5 in the Supplement).

## 4. Discussion

In this cross-sectional study, we found that a low-carbohydrate diet was associated with a lower risk of frailty and pre-frailty, whereas a low-fat diet was not associated with a lower risk of frailty and pre-frailty in community-dwelling aging Chinese adults.

To date, numerous studies have examined the associations between dietary macronutrients and frailty status, but controversies exist regarding the optimal amounts of macronutrient intake in preventing frailty. A longitudinal Rotterdam study suggested that total carbohydrate and total fat were not associated with frailty, whereas high animal protein intake was positively linked to frailty [26]. However, a cross-sectional study from the same Rotterdam study found that neither specifically plant nor animal protein intake was associated with lower frailty [27]. Another cross-sectional study indicated no significant reduction in the risk of pre-frailty/frailty with higher protein intakes [28]. However, two prospective cohort studies confirmed that daily protein intake was inversely associated with the risk of pre-frailty/frailty in older adults [29,30]. However, a meta-analysis based on observational studies indicated that absolute or adjusted protein intake, or protein intake relative to total energy intake, were not significantly associated with frailty in older adults [31]. Therefore, the authors considered that several protein-related parameters combined with the source of protein may be more important than protein amount alone [32]. Additionally, a longitudinal study, the Baltimore Longitudinal Study of Aging, described an association between high total carbohydrate intake and increased frailty risk [15]. A 15-year follow-up study in France showed that higher intake of simple carbohydrates was significantly associated with a greater risk of frailty defined using the frail phenotype, but no associations were observed for complex or total carbohydrate intake or low-carbohydrate diets [20]. However, our study found that high total carbohydrate intake was significantly associated with a greater risk of frailty (Q4 vs. Q1 (adjusted OR = 1.87; 95% CI = 1.25–2.78)). The inconsistent results may be attributed to differences in average carbohydrate intake (209 g/day in this study population vs. 235 g/day in our study); lower total carbohydrate intake coincides with reduced dietary fiber intake and dietary fiber was related to reduced frailty risk [15,33]. Thus, the source of high-quality carbohydrates is essential for frailty prevention. These existing studies focused on single or specific macronutrient intakes without considering the impact of percentages of energy from each macronutrient. Importantly, the biggest health benefits will be obtained from combinations of macronutrients [31]. In the current study, we incorporated LCD and LFD scores, which combined the percentages of energy from three macronutrients, to reflect daily dietary pattern and quality. Our results indicated that the LCD score, not the LFD score, was associated with lower frailty and pre-frailty status. Although the roles of LCD and LFD scores in health outcomes have been widely studied, the evidence is limited regarding associations between LCD and LFD scores with frailty risk in aging populations. Only one study defined a low-carbohydrate diet as total daily energy intake from carbohydrates ≤ 45%; the findings showed no association between a low-carbohydrate diet and frailty [20]. The conflict with our results may be explained by differences in frailty definitions. Our study defined frailty using the FI, whereas the other study used the frailty phenotype. The FI identifies more individuals as frail due to cumulative effects [34,35]. In other words, the results also depend on the frailty assessment tool used [36]. Moreover, different definitions of low-carbohydrate diet may affect the results; we analyzed results using the same defining criteria (≤45% of energy) and found no association of a low-carbohydrate diet defined as such with pre-frailty (adjusted OR = 0.97; 95%CI = 0.86–1.09) and frailty (adjusted OR = 1.01; 95% CI = 0.84–1.21). Age differences in study participants or the study design may also partly explain the differences. Likewise, a cross-sectional study of 954 older Korean adults found that carbohydrate intake above the acceptable macronutrient distribution range (65% of energy) was positively related to higher frailty risk [37]. Therefore, our study extends the current evidence that the LCD score representing carbohydrate restriction could influence frailty in the elderly. The potential mechanisms linking lower carbohydrate intake with lower FI are largely unknown. The possible mechanisms are that diets low in carbohydrate may decrease chronic inflammation, oxidative stress [38] and insulin resistance [39], which in turn may decrease the risk of developing chronic diseases associated with frailty.

A low-fat diet has been widely advocated for preventing obesity and cardiovascular diseases. However, there is a scarcity of evidence on the topic of LFD score and frailty. In this study, the LFD score was not associated with lower frailty or pre-frailty, and SFA, MUFA and PUFA did not affect frailty status. However, in another cross-sectional survey in China, a low-fat diet was positively correlated with frailty risk [40]. Among older Japanese participants, higher meat and fat intake were negatively associated with the development of frailty [41]. High energy intake from fat was related to a decreased prevalence of frailty [37]. In addition, some unhealthy animal fats like red meat consumption were associated with a higher risk of frailty, while fish, nuts and low-fat dairy reduced the risk of frailty [42]. A high-fat, low-fiber diet pattern was a risk factor for frailty in British populations [43]. Notably, although low-fat diets aim to lower the fat content of the average diet or reduce the percentage of calories from fat, there is currently no uniform definition of a low-fat diet. The LFD score was defined based on the energy contributions of fat restriction in our study. Further studies are needed to explore the influence of LFD score and different qualities and sources of fat on frailty in different populations.

To our knowledge, this is the first epidemiological study to comprehensively examine associations between dietary nutrition and frailty risks in Chinese populations, which provides an opportunity to interpret the role of macronutrients in frailty. Several limitations in our study should be acknowledged. First, as with all cross-sectional studies, we were unable to establish causal relationships between diet scores and frailty. In particular, individuals with diabetes and obesity were more compliant with medical recommendations to change their diets during the study period, which may have affected these relationships, though we did carefully adjust for multiple confounders and performed hierarchical analysis to reduce this possibility. Second, we used an FFQ to assess self-reported dietary intake over the past 12 months, so recall bias may exist. However, our previous research confirmed acceptable consistency between FFQs and 24 h dietary recall among adults in rural Ningxia [44]. Third, our findings may have limited generalizability because the study participants were from rural areas of northwest China. The types of foods consumed were generally lacking diversity, and the main energy sources were rice and noodles. Fruit and root vegetable intake was relatively low in the elderly people in these areas; therefore, the results may not fully represent the diet quality of the entire population. Finally, the scoring criteria of the LCD and LFD scores were based on ranking the percentages of energy from total macronutrients, and the scores were not converted to standards for measuring diet directly.

## 5. Conclusions

In this cross-sectional study of older Chinese adults, a low-carbohydrate diet was significantly associated with a lower risk of frailty or pre-frailty, whereas a lower-fat diet was not significantly related to frailty risk in our study population. These findings suggest that adopting relatively lower-carbohydrate eating patterns may help reduce the risk of frailty and pre-frailty among older adults.

## Figures and Tables

**Table 1:** Characteristics of the study participants by frailty status.

Variables	Total (*n* = 6414)	Robust (*n* = 3152)	Pre-Frail (*n* = 2475)	Frail (*n* = 787)	*p* Value
Age (years)	66.60 ± 4.15	66.24 ± 4.09	66.87 ± 4.25	67.208 ± 3.99	<0.001
Age (years), *n* (%)					<0.001
60–64	2205 (34.4)	1211 (38.4)	784 (31.7)	210 (26.7)	
65–69	2602 (40.5)	1224 (38.8)	1031 (41.7)	347 (44.1)	
70–74	1403 (21.9)	639 (20.3)	558 (22.5)	206 (26.2)	
≥75	204 (3.2)	78 (2.5)	102 (4.1)	24 (3.0)	
Sex, *n* (%)					<0.001
Male	3065 (47.8)	1645 (52.2)	1149 (46.4)	271 (34.4)	
Female	3349 (52.2)	1507 (47.8)	1326 (53.6)	516 (65.6)	
Education level *n* (%)					<0.001
Primary school or lower	5611 (87.4)	2732 (86.6)	2141 (86.5)	738 (93.8)	
Junior high school	668 (10.4)	339 (10.8)	287 (11.6)	42 (5.3)	
Senior high school or above	135 (2.2)	81 (2.6)	47 (1.9)	7 (0.9)	
Current smoker, *n* (%)					<0.001
Yes	927 (14.5)	519 (16.5)	339 (13.7)	69 (8.8)	
No	5487 (85.5)	2633 (83.5)	2136 (86.3)	718 (91.2)	
Current drinker, *n* (%)					<0.001
Yes	1323 (20.6)	679 (21.5)	550 (22.2)	94 (11.9)	
No	5091 (79.4)	2473 (78.5)	1925 (77.8)	693 (88.1)	
WC (cm)	86.22 ± 9.85	83.61 ± 9.30	88.15 ± 9.57	90.60 ± 9.95	<0.001
WHR	0.92 ± 0.66	0.90 ± 0.06	0.93 ± 0.06	0.95 ± 0.07	<0.001
BMI (kg/m^2^)	24.88 ± 3.46	23.99 ± 3.28	25.54 ± 3.33	26.36 ± 3.59	<0.001
SBP (mmHg)	141 ± 20	130 ± 16	151 ± 17	150 ± 18	<0.001
DBP (mmHg)	84 ± 12	78 ± 11	89 ± 12	89 ± 12	<0.001
FBG (mmol/L)	5.96 ± 5.26	5.92 ± 5.40	5.87 ± 2.15	6.42 ± 4.27	0.006
TG (mmol/L)	1.65 ± 1.12	1.53 ± 1.05	1.74 ± 1.18	1.84 ± 1.20	<0.001
TC (mmol/L)	4.88 ± 1.49	4.81 ± 1.05	4.94 ± 1.99	4.97 ± 1.10	0.001
HDL-C (mmol/L)	1.31 ± 0.35	1.33 ± 0.35	1.29 ± 0.35	1.29 ± 0.31	<0.001
LDL-C (mmol/L)	2.88 ± 0.87	2.87 ± 0.87	2.85 ± 0.87	3.01 ± 0.90	0.001
LCD score	14.4 ± 8.4	14.7 ± 8.4	14.0 ± 8.4	14.1 ± 8.7	0.006
LFD Score	10.4 ± 8.3	10.5 ± 8.3	10.1 ± 8.2	10.9 ± 8.4	0.047
Energy (kcal/d)	1957 ± 650	1974 ± 674	1949± 638	1912 ± 585	0.046
Total carbohydrate (g/d)	235 ± 79	236 ± 82	235 ± 78	230 ± 77	0.098
Total protein (g/d)	63 ± 37	65 ± 38	61 ± 35	63 ± 37	<0.001
Total fat (g/d)	77 ± 36	77 ± 36	77 ± 36	75 ± 35	0.227
Total carbohydrate (% of energy)	48.9 ± 8.6	48.8 ± 8.5	49.1 ± 8.7	48.8 ± 9.0	0.415
Total protein (% of energy)	12.5 ± 4.4	12.7 ± 4.5	12.2 ± 4.3	12.7 ± 4.7	<0.001
Total fat (% of energy)	35.0 ± 9.9	34.9 ± 9.8	35.2 ± 9.9	34.9 ± 10.3	0.404
SFA (g/d)	12 ± 6	12 ± 7	12 ± 6	12 ± 6	0.292
MUFA (g/d)	26 ± 12	26 ± 13	27 ± 12	26 ± 12	0.159
PUFA (g/d)	28 ± 16	28 ± 16	28 ± 16	27 ± 16	0.540

Values are mean ± standard deviation for continuous variables and number (%) for categorical variables. Abbreviation: WC, waist circumference; WHR, waist-to-hip ratio; BMI, body mass index; SBP, systolic blood pressure; DBP, diastolic blood pressure; FBG, fasting blood glucose; TG, triacylglycerols; TC, total cholesterol; HDL, high-density lipoprotein; LDL, low-density lipoprotein; LCD, low-carbohydrate diets; LFD, low-fat-diets; SFA, saturated fatty acid; MUFA, monounsaturated fatty acid; PUFA, polyunsaturated fatty acid.

**Table 2:** Frail status and characteristics of study participants according to quartiles of LCD score and LFD score.

Variables	LCD Score					LFD Score				
	Q1 (<8)	Q2 (8–17)	Q3 (18–20)	Q4 (≥21)	*p* Trend	Q1 (<1)	Q2 (1–10)	Q3 (11–19)	Q4 (≥20)	*p* Trend
Robust *n* (%)	726 (45.7)	906 (50.4)	960 (48.2)	560 (53.9)	0.003	646 (48.9)	1074 (49.2)	751 (48.0)	681 (50.6)	0.607
Prefrail *n* (%)	635 (40.0)	700 (39.0)	792 (39.8)	348 (33.5)		533 (40.3)	856 (39.2)	584 (37.3)	502 (37.3)	
Frail *n* (%)	228 (14.3)	191 (10.6)	238 (12.0)	130 (12.5)		142 (10.8)	252 (11.6)	230 (14.7)	163 (12.1)	
WC (cm)	86.66 ± 9.93	86.37 ± 9.87	85.87 ± 9.81	85.96 ± 9.76	0.035	85.63 ± 9.78	86.20 ± 9.88	86.39 ± 9.99	86.64 ± 9.71	0.007
WHR	0.92 ± 0.07	0.92 ± 0.07	0.91 ± 0.07	0.91 ± 0.06	0.062	0.91 ± 0.07	0.92 ± 0.07	0.92 ± 0.07	0.92 ± 0.07	0.005
BMI (kg/m^2^)	25.07 ± 3.50	24.93 ± 3.48	24.76 ± 3.41	24.72 ± 3.46	0.004	24.72 ± 3.34	24.84 ± 3.48	24.98 ± 3.56	24.97 ± 3.44	0.035
SBP (mmHg)	142 ± 20	141 ± 20	142 ± 20	137 ± 18	<0.001	142 ± 20	142 ± 20	139 ± 20	140 ± 20	0.004
DBP (mmHg)	84 ± 12	84 ± 13	84 ± 13	82 ± 11	<0.001	83 ± 13	84 ± 12	83 ± 12	84 ± 12	0.940
FBG (mmol/L)	6.22 ± 6.21	5.81 ± 2.81	5.95 ± 3.78	5.86 ± 3.46	0.076	5.86 ± 3.06	5.97 ± 4.22	6.21 ± 6.23	5.77 ± 1.85	0.964
TG (mmol/L)	1.61 ± 1.00	1.70 ± 1.21	1.60 ± 1.15	1.71 ± 1.08	0.135	1.55 ± 1.13	1.65 ± 1.13	1.68 ± 1.08	1.70 ± 1.15	0.001
TC (mmol/L)	4.86 ± 1.00	4.94 ± 1.70	4.87 ± 1.76	4.81 ± 1.12	0.235	4.85 ± 1.99	4.91 ± 1.13	4.89 ± 1.77	4.84 ± 1.03	0.834
HDL-C (mmol/L)	1.30 ± 0.32	1.34 ± 0.36	1.31 ± 0.36	1.30 ± 0.35	0.366	1.30 ± 0.38	1.34 ± 0.32	1.31 ± 0.37	1.29 ± 0.31	0.071
LDL-C (mmol/L)	2.78 ± 0.81	2.95 ± 0.86	2.81 ± 0.88	3.07 ± 0.93	<0.001	2.70 ± 0.82	2.94 ± 0.89	2.94 ± 0.89	2.90 ± 0.84	<0.001
Energy (kcal/d)	1761 ± 647	1849 ± 612	2030 ± 575	2303 ± 694	<0.001	2026 ± 569	1952 ± 681	2010 ± 641	1833 ± 667	<0.001
Total carbohydrate (g/d)	260 ± 94	235 ± 75	212 ± 64	240 ± 75	<0.001	198 ± 57	222 ± 68	259 ± 80	265 ± 94	<0.001
Total protein (g/d)	51 ± 22	58 ± 30	54 ± 29	110 ± 44	<0.001	47 ± 22	63 ± 38	73 ± 40	69 ± 38	<0.001
Total fat (g/d)	49 ± 20	64 ± 20	101 ± 36	97 ± 35	<0.001	111 ± 36	81 ± 33	66 ± 23	49 ± 21	<0.001
Total carbohydrate (% of energy)	59.3 ± 4.2	51.3 ± 4.1	42.1 ± 5.8	41.9 ± 5.0	<0.001	39.4 ± 5.0	46.5 ± 6.1	52.1 ± 5.3	58.5 ± 5.9	<0.001
Total protein (% of energy)	11.4 ± 1.8	12.2 ± 3.6	10.5 ± 3.8	18.9 ± 4.0	<0.001	9.0 ± 2.2	12.4 ± 3.8	14.1 ± 4.9	14.5 ± 4.3	<0.001
Total fat (% of energy)	25.0 ± 4.0	31.7 ± 4.9	44.6 ± 8.7	37.8 ± 6.0	<0.001	49.0 ± 6.3	37.4 ± 4.9	29.6 ± 3.6	23.8 ± 3.4	<0.001
SFA (g/d)	7.5 ± 3.3	9.9 ± 4.1	14.0 ± 5.7	18.6 ± 7.8	<0.001	14.6 ± 6.0	12.8 ± 7.4	11.4 ± 5.5	8.9 ± 4.9	<0.001
MUFA (g/d)	16.9 ± 6.9	21.9 ± 7.2	34.3 ± 12.3	33.4 ± 12.7	<0.001	37.8 ± 12.5	28.0 ± 11.6	22.7 ± 8.0	16.9 ± 7.2	<0.001
PUFA (g/d)	15.9 ± 7.9	22.4 ± 8.6	40.3 ± 17.2	32.5 ± 14.5	<0.001	46.0 ± 16.9	29.8 ± 12.5	21.8 ± 8.8	14.2 ± 7.2	<0.001

Values are means ± SDs for continuous variables and *n* (%) for categorical variables. Abbreviation: Q1–Q4: Quartile 1–Quartile 4. The abbreviations for the other variables are the same as in Table 1.

**Table 3:** Multivariate-adjusted odds ratio (ORs) and 95% confidence intervals (CIs) of pre-frailty or frailty according to quartile dietary fat.

	Robust/Pre-Frail total *n*/*n*	Pre-Frail OR (95%CI)		Robust/Frail Total *n*/*n*	Frail OR (95%CI)	
		Model 1	Model 2		Model 1	Model 2
**LCD score**						
Q1	1361 (726/635)	1.00 (Ref)	1.00 (Ref)	954 (726/228)	1.00 (Ref)	1.00 (Ref)
Q2	1606 (906/700)	0.88 (0.76–1.02)	0.88 (0.76–1.03)	1097 (906/191)	0.68 (0.55–0.84)	0.65 (0.52–0.82)
Q3	1752 (960/792)	0.93 (0.81–1.08)	0.96 (0.82–1.11)	1198 (960/238)	0.78 (0.63–0.96)	0.85 (0.68–1.06)
Q4	908 (560/348)	0.71 (0.60–0.84)	0.73 (0.61–0.88)	690 (560/130)	0.74 (0.58–0.94)	0.73 (0.55–0.95)
*p* for trend		0.003	0.017		0.008	0.035
**LFD score**						
Q1	1179 (646/533)	1.00 (Ref)	1.00 (Ref)	788 (646/142)	1.00 (Ref)	1.00 (Ref)
Q2	1930 (1074/856)	0.98 (0.84–1.13)	0.96 (0.83–1.12)	1326 (1074/252)	1.10 (0.88–1.38)	1.04 (0.82–1.33)
Q3	1335 (751/584)	0.96 (0.82–1.12)	0.95 (0.81–1.12)	981 (751/230)	1.43 (1.13–1.81)	1.31 (1.02–1.68)
Q4	1183 (681/502)	0.92 (0.78–1.08)	0.89 (0.75–1.05)	844 (681/163)	1.12 (0.87–1.44)	1.01 (0.77–1.32)
*p* for trend		0.291	0.203		0.039	0.275

Model 1: Age-adjusted. Model 2: adjusted for age (years), sex (male, female), educational status (primary school or lower, junior high school, senior high school or above), cigarette smoking (never smoked, smoking every day, occasional smoking), drinking (yes, no), physical activity (never, 1–2 times/week, 3–5 times/week, everyday), BMI (quartile) and total energy intake (quartile). Abbreviation: LCD, low-carbohydrate diet; LFD, low-fat-diet; Q1–Q4: Quartile 1–Quartile 4.

## Data Availability

Data are available from the corresponding author upon reasonable request.

## Supplementary Materials

The following supporting information can be downloaded at https://www.mdpi.com/article/10.3390/nu15143084/s1, Table S1: List of 28 variables used to construct frailty index; Table S2: Criteria for determining the low-carbohydrate diet (LCD) score; Table S3: Criteria for determining the low-fat diet (LFD) score; Table S4: Multivariate-adjusted odds ratio (ORs) and 95% confidence intervals (Cis) of pre-frailty or frailty according to quartile of dietary fatty acid. Table S5. Multivariate-adjusted odds ratio (ORs) and 95% confidence intervals (CIs) of pre-frail or frailty according to LCD and LFD by sex. Figure S1: Stratified analysis of LCD and LFD scores with pre-frailty and frailty by participants’ characteristics.
