# Supplementary material for: Associations between Low-Carbohydrate Diets and Low-Fat Diets with Frailty in Community-Dwelling Aging Chinese Adults
Source: Nutrients. 2023 Jul 9;15(14):3084. doi: 10.3390/nu15143084 (PMC10383029; doi:10.3390/nu15143084)
Supplement: Supplementary file 1 [file nutrients-15-03084-s001.zip › nutrients-2464763-supplementary.pdf]

Table S1. List of 28 variables used to construct frailty index

	Definition according to self-report, physical measurements, or both	Coding of variables
1	Self-reported diagnosis of hypertension by a doctor, self-reported use of antihypertension drugs, systolic blood pressure measured to be ≥140 mm Hg, or diastolic blood pressure measured to be ≥90 mm Hg	Yes=1; no=0
2	Self-reported diagnosis of heart disease by a doctor	Yes=1; no=0
3	Self-reported diagnosis of stroke or transient ischaemic attack by a doctor	Yes=1; no=0
4	Self-reported diagnosis of emphysema or chronic bronchitis by a doctor	Yes=1; no=0
5	Self-reported diagnosis of tuberculosis by a doctor	Yes=1; no=0
6	Self-reported diagnosis of asthma by a doctor	Yes=1; no=0
7	Self-reported diagnosis of peptic ulcer by a doctor	Yes=1; no=0
8	Self-reported diagnosis of gallstone disease or cholecystitis by a doctor	Yes=1; no=0
9	Self-reported diagnosis of rheumatoid arthritis by a doctor	Yes=1; no=0
10	Self-reported diagnosis of fracture by a doctor	Yes=1; no=0
11	Self-reported diagnosis of neurasthenia by a doctor	Yes=1; no=0
12	Self-reported diagnosis of diabetes, fasting blood glucose measured to be ≥7·0 mmol/L, or random blood glucose measured to be ≥11·1 mmol/L	Yes=1; no=0
13	Self-reported diagnosis of cancer by a doctor	Yes=1; no=0
14	Self-reported diagnosis of chronic kidney disease by a doctor	Yes=1; no=0
15	If you were walking on level ground with other healthy people of the same age, would you usually become short of breath or slow down because of chest discomfort?	Yes=1; no=0
16	During the past month, did you have any of the following for ≥3 days per week: (1) taking >30 min to fall asleep after going to bed or waking up in the middle of the night; (2) waking up early and not being able to go back to sleep; or (3) having difficulty staying alert while at work, eating, or meeting people during the daytime?	Yes=1; no=0
17	How often do you have bowel movements each week?	<3 times per week=1; other=0
18	During the past 12 months, did you have pain or discomfort in your body lasting ≥3 months that interfered with your life?	Yes=1; no=0
19	During the past 12 months, have you developed a frequent cough?	Yes, for ≥3 months=1; yes, for <3 months=0.5; no=0
20	Do you brush your teeth rarely or never, or have false teeth?	Yes=1; no=0
21	During the past 12 months, have you lost weight (≥2·5 kg) despite not trying to intentionally lose weight?	Yes=1; no=0
22	During the past 12 months, did you feel much sadder, or more depressed, than usual for ≥2 weeks?	Yes=1; no=0
23	How is your current general health status?	Poor=1; fair=0.5; good=0.25; excellent=0
24	Body-mass index (kg/m^2)	<18·5 or ≥28.0=1; ≥24.0 and <28.0=0.50; ≥18.5 and

		<24.0=0
25	Waist circumference (cm) to hip circumference ratio	⩾0.95 for men or ≥0.90 for women=1; ≥0.90 and <0.95 for men or ≥0.85 and <0.90 for women=0.50; <0.90 for men or <0.85 for women=0
26	Measured heart rate, beats per min	<60 or >100=1; ≥60 and ≤100=0
27	Self-reported diagnosis of osteoporosis by a doctor	Yes=1; no=0
28	How often do you participate in physical activity in your spare time in the past 12 months	<3 times per week=1; ≥3 times per week=0

Table S2. Criteria for Determining the Low-Carbohydrate-Diet (LCD) Score

Points	Total Carbohydrate	Total Protein	Total Fat
	Percentage of energy		
0	>57.3	<14.2	<26.9
1	55.4-58.7	13.6-14.8	26.0-28.6
2	54.2-57.0	14.2-15.3	27.6-29.8
3	53.1-55.7	14.7-15.7	28.7-30.7
4	52.2-54.7	15.1-16.1	29.7-31.6
5	51.2-53.7	15.5-16.4	30.6-32.5
6	50.2-52.8	15.9-16.8	31.5-33.4
7	49.0-51.8	16.3-17.3	32.3-34.3
8	47.6-50.6	16.7-17.9	33.3-35.5
9	45.5-49.3	17.3-18.7	34.5-37.4
10	<47.3	>18.1	>36.3

Note: LCD score is the sum of the scores for the three nutrients, ranging from 0 to 30.

Table S3. Criteria for Determining the Low-Fat-Diet(LFD) Score

Points	Total Fat	Total Carbohydrate	Total Protein
	Percentage of energy		
0	>36.3	<47.3	<14.2
1	34.5-37.4	45.5-49.3	13.6-14.8
2	33.3-35.5	47.6-50.6	14.2-15.3
3	32.3-34.3	49.0-51.8	14.7-15.7
4	31.5-33.4	50.2-52.8	15.1-16.1
5	30.6-32.5	51.2-53.7	15.5-16.4
6	29.7-31.6	52.2-54.7	15.9-16.8
7	28.7-30.7	53.1-55.7	16.3-17.3
8	27.6-29.8	54.2-57.0	16.7-17.9
9	26.0-28.6	55.4-58.7	17.3-18.7
10	<26.9	>57.3	>18.1

Note: LFD score is the sum of the scores for the three nutrients, ranging from 0 to 30.

Table S4. Multivariate-adjusted odds ratio (ORs) and 95% confidence intervals (CIs) of pre-frail or frailty according to quartile of dietary fatty acid

	Pre-frailty OR (95%CI)		Frailty OR (95%CI)	
	**Model 1**	**Model 2**	**Model 1**	**Model 2**
**SFAs**				
Q1	1.00 (Ref)	1.00 (Ref)	1.00 (Ref)	1.00 (Ref)
Q2	0.93 (0.80-1.08)	1.00 (0.84-1.19)	1.01 (0.81-1.27)	1.27 (0.97-1.66)
Q3	1.00 (0.86-1.16)	1.05 (0.84-1.30)	1.45 (1.17-1.80)	1.72 (1.25-2.37)
Q4	0.97 (0.84-1.13)	1.04 (0.80-1.34)	0.86 (0.68-1.08)	1.16 (0.78-1.74)
P for trend	1.000	1.00	0.999	1.00
**MUFAs**				
Q1	1.00 (Ref)	1.00 (Ref)	1.00 (Ref)	1.00 (Ref)
Q2	0.89 (0.76-1.04)	0.93 (0.78-1.09)	1.04 (0.83-1.29)	1.05 (0.82-1.34)
Q3	0.92 (0.79-1.08)	0.95 (0.78-1.15)	0.78 (0.62-0.99)	0.76 (0.57-1.02)
Q4	1.01 (0.86-1.18)	1.06 (0.84-1.33)	0.87 (0.69-1.10)	0.85 (0.60-1.19)
P for trend	0.507	1.00	0.077	0.992
**PUFAs**				
Q1	1.00 (Ref)	1.00 (Ref)	1.00 (Ref)	1.00 (Ref)
Q2	0.88 (0.76-1.02)	0.96 (0.83-1.12)	0.92 (0.74-1.14)	0.98 (0.78-1.24)
Q3	0.99 (0.86-1.15)	0.95 (0.81-1.12)	0.88 (0.71-1.10)	0.91 (0.71-1.17)
Q4	0.91 (0.79-1.06)	0.89 (0.75-1.05)	0.85 (0.68-1.06)	0.94 (0.72-1.25)
P for trend	0.587	0.999	0.159	0.998

Model 1: Age-adjusted. Model 2: adjusted for age (years), sex (male, female), educational status(primary school or lower, junior high school, senior high school or above), cigarette smoking(never smoked, smoking every day, occasional smoking), drinking(yes, no), physical activity (never, 1-2 times/week, 3-5 times/week, everyday), BMI(quartile) and total energy intake(quartile).

Abbreviation: SFAs saturated fatty acids; MUFAs monounsaturated fatty acids; PUFAs poly unsaturated fatty acids; Q1-Q4: Quartile 1-Quartile 4.

Table S5. Multivariate-adjusted odds ratio (ORs) and 95% confidence intervals (CIs) of pre-frail or frailty according to LCD and LFD by sex*

	**Male(n=3065)**		**Females(n=3349)**	
	**Pre-frailty(n=1149)**	**Frailty(n=271)**	**Pre-frailty(n=1329)**	**Frailty(n=516)**
	**OR (95%CI)**	**OR (95%CI)**	**OR (95%CI)**	**OR (95%CI)**
**LCD score**				
Q1	1.00 (Ref)	1.00 (Ref)	1.00 (Ref)	1.00 (Ref)
Q2	0.85 (0.68-1.06)	0.63 (0.43-0.92)	0.91 (0.74-1.13)	0.66 (0.49-0.89)
Q3	1.09 (0.88-1.35)	0.87 (0.60-1.22)	0.87 (0.70-1.08)	0.85 (0.63-1.14)
Q4	0.91 (0.69-1.18)	0.90 (0.58-1.40)	0.62 (0.47-0.80)	0.65 (0.46-0.92)
P for trend	0.934	0.450	0.002	0.044
**LFD score**				
Q1	1.00 (Ref)	1.00 (Ref)	1.00 (Ref)	1.00 (Ref)
Q2	0.81 (0.65-1.01)	0.99 (0.67-1.48)	1.09 (0.88-1.35)	1.04 (0.76-1.42)
Q3	0.85 (0.67-1.07)	1.15 (0.77-1.74)	1.03 (0.81-1.30)	1.36 (0.98-1.87)
Q4	0.84 (0.66-1.07)	0.86 (0.56-1.34)	0.89 (0.67-1.14)	1.11 (0.78-1.56)
P for trend	0.254	0.866	0.310	0.148

*adjusted for age (years), educational status(primary school or lower, junior high school, senior high school or above), cigarette smoking(never smoked, smoking every day, occasional smoking), drinking(yes, no), physical activity (never, 1-2 times/week, 3-5 times/week, everyday), BMI(quartile) and total energy intake(quartile).

Abbreviation: LCD low-carbohydrate diets; LFD Low-fat-diets; Q1-Q4: Quartile 1-Quartile 4

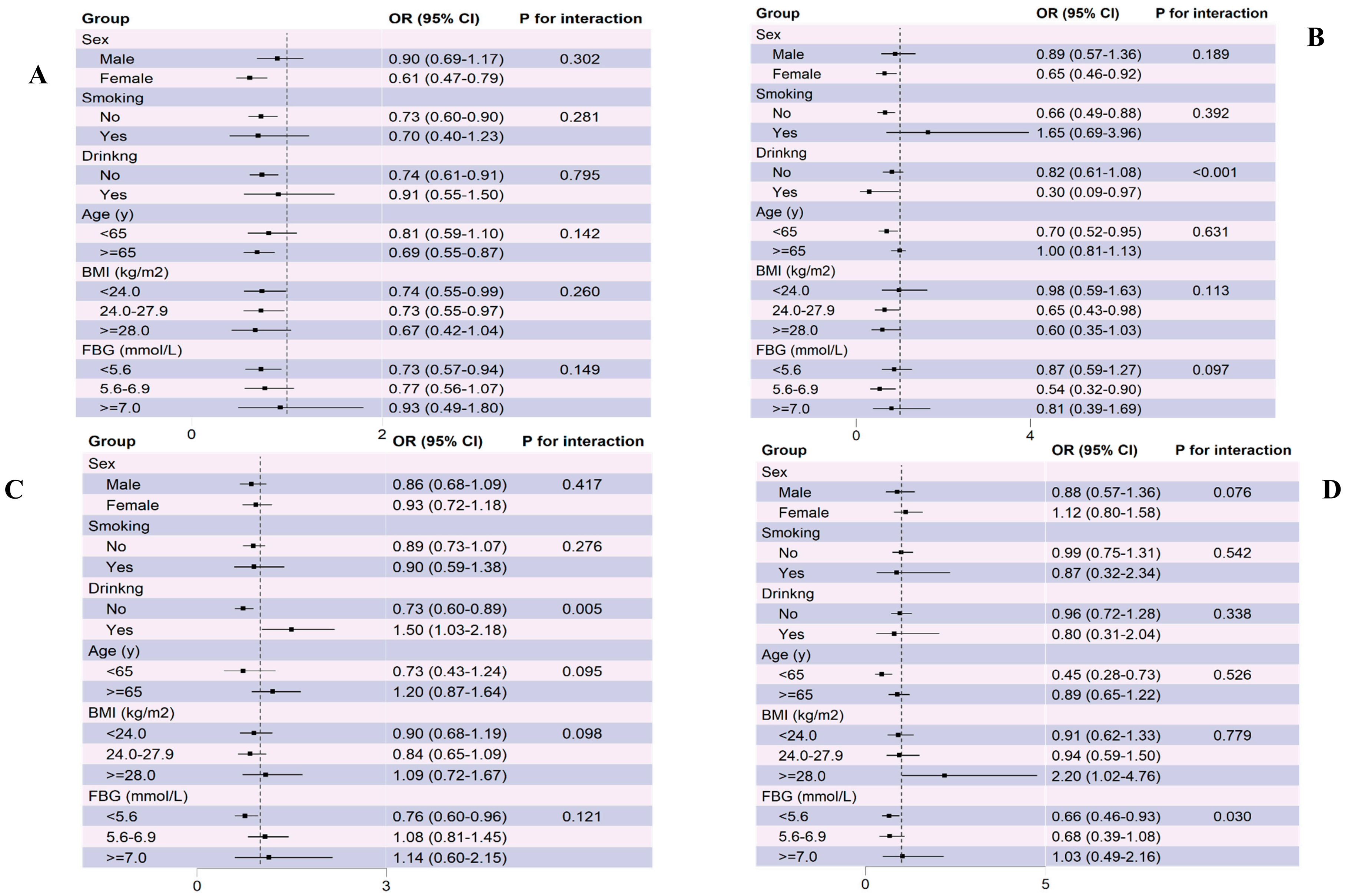

Figure S1 Stratified analysis of LCD and LFD scores with pre-frail and frail by participants' characteristics. The odds ratios and 95% confidence intervals were calculated using logistic regression models after multivariate-adjusted. A: pre-frail with LCD; B: frail with LCD; C: pre-frail with LFD; D: frail with LFD.
